# Supplementary material for: Genomic diversity of Neisseria gonorrhoeae Isolates in Kenya revealed by MLST, NG-MAST, and NG-STAR typing
Source: PLoS One. 2026 May 19;21(5):e0335831. doi: 10.1371/journal.pone.0335831 (PMC13186387; doi:10.1371/journal.pone.0335831)
Supplement: S1 Table — (DOCX) [file pone.0335831.s001.docx]

**S1 Table. Isolate metadata and antimicrobial susceptibility data.**

|  |  | **Isolate details** | | | **Metadata** | | **MICs (mg/L)** | | | | | | |
| --- | --- | --- | --- | --- | --- | --- | --- | --- | --- | --- | --- | --- | --- |
|  | **Sample ID** | **PubMLST IDs** | **Year of isolation** | **Region** | **Sex** | **Age** | **CFX** | **CRO** | **PEN** | **TET** | **CIP** | **AZM** | **SPT** |
| 1 | KNY_NGAMR1 | 60436 | 2015 | Nairobi | Male | >18 | <0.016 | <0.002 | 0.064 | 0.064 | 0.38 | 0.25 | 8 |
| 2 | KNY_NGAMR2 | 60501 | 2015 | Nairobi | Male | 20-29 | <0.016 | <0.002 | 64 | 0.5 | 0.016 | 0.5 | 8 |
| 3 | KNY_NGAMR3 | 60502 | 2015 | Nairobi | Male | 20-29 | <0.016 | NT | 48 | 16 | 3 | 0.125 | 6 |
| 4 | KNY_NGAMR4 | 60503 | 2016 | Coast | Male | 30-39 | <0.016 | <0.002 | 8 | 12 | 0.006 | 0.125 | 2 |
| 5 | KNY_NGAMR5 | 60504 | 2016 | Nyanza | Male | 18-19 | <0.016 | 0.006 | 3 | 16 | 4 | 0.125 | 2 |
| 6 | KNY_NGAMR6 | 60505 | 2017 | Coast | Male | 20-29 | <0.016 | <0.016 | 0.094 | 0.75 | 12 | 0.25 | 3 |
| 7 | KNY_NGAMR7 | 60506 | 2014 | Coast | Male | 18-19 | <0.016 | 0.008 | >256 | 16 | 8 | 2 | 16 |
| 8 | KNY_NGAMR8 | 60507 | 2013 | Nyanza | Female | 18-19 | <0.016 | 0.002 | 0.064 | 0.5 | 3 | 0.25 | 4 |
| 9 | KNY_NGAMR9 | 59511 | 2016 | Nyanza | Male | 20-29 | <0.016 | <0.016 | 0.094 | 0.32 | 12 | 0.25 | 6 |
| 10 | KNY_NGAMR10 | 60508 | 2016 | Nyanza | Male | 30-39 | <0.016 | <0.016 | >256 | 32 | 8 | 0.38 | 16 |
| 11 | KNY_NGAMR11 | 60509 | 2016 | Nyanza | Male | 30-39 | <0.016 | <0.016 | 12 | 12 | 3 | 0.25 | 8 |
| 12 | KNY_NGAMR13 | 60519 | 2014 | Rift Valley | Male | 20-29 | <0.016 | <0.002 | 8 | 12 | 3 | 0.125 | 24 |
| 13 | KNY_NGAMR14 | 60511 | 2015 | Rift Valley | Male | 20-29 | <0.016 | <0.002 | 64 | 24 | 16 | 0.125 | 8 |
| 14 | KNY_NGAMR15 | 60516 | 2014 | Nyanza | Male | 20-29 | <0.016 | <0.016 | 48 | 32 | 4 | 0.5 | 12 |
| 15 | KNY_NGAMR16 | 61330 | 2015 | Nyanza | Male | 30-39 | <0.016 | <0.016 | 0.19 | 4 | 8 | 0.38 | 4 |
| 16 | KNY_NGAMR17 | 60513 | 2015 | Nyanza | Male | 20-29 | <0.016 | <0.016 | 0.38 | 16 | 6 | 0.5 | 6 |
| 17 | KNY_NGAMR18 | 60514 | 2015 | Nyanza | Male | 20-29 | <0.016 | <0.016 | 0.5 | 24 | 0.38 | 0.5 | 6 |
| 18 | KNY_NGAMR19 | 60515 | 2015 | Nyanza | Male | 20-29 | <0.016 | 0.094 | 0.19 | 64 | 4 | 1.5 | 1 |
| 19 | KNY_NGAMR20 | 60512 | 2015 | Nyanza | Male | 30-39 | <0.016 | 0.004 | 12 | 24 | 8 | 0.125 | 12 |
| 20 | KNY_NGAMR21 | 60517 | 2016 | Nyanza | Male | 30-39 | <0.016 | <0.016 | 32 | 24 | 3 | 0.125 | 12 |
| 21 | KNY_NGAMR22 | 60518 | 2016 | Nyanza | Male | 20-29 | <0.016 | 0.004 | 2 | 32 | 4 | 1 | 12 |
| 22 | KNY_NGAMR23 | 60510 | 2017 | Nyanza | Female | 30-39 | <0.016 | <0.002 | 12 | 8 | 12 | 0.125 | 4 |
| 23 | KNY_NGAMR24 | 60520 | 2014 | Nyanza | Male | 20-29 | <0.016 | <0.016 | 2 | 16 | 4 | 0.38 | 8 |
| 24 | KNY_NGAMR26 | 60439 | 2016 | Nyanza | Male | 20-29 | <0.016 | <0.016 | 8 | 32 | 1.5 | 0.25 | 8 |
| 25 | KNY_NGAMR28 | 60521 | 2017 | Nyanza | Female | 20-29 | <0.016 | <0.002 | 96 | 32 | 24 | 0.5 | 8 |
| 26 | KNY_NGAMR29 | 60522 | 2017 | Nyanza | Male | 20-29 | <0.016 | <0.002 | 0.094 | 12 | 12 | 0.25 | 2 |
| 27 | KNY_NGAMR30 | 60441 | 2017 | Nyanza | Male | 18-19 | <0.016 | <0.002 | 12 | 12 | 4 | 0.125 | 8 |
| 28 | KNY_NGAMR31 | 60523 | 2017 | Nyanza | Male | 18-19 | <0.016 | <0.002 | 0.38 | 48 | >32 | 0.25 | 3 |
| 29 | KNY_NGAMR32 | 60524 | 2017 | Nyanza | Female | 20-29 | <0.016 | <0.002 | 8 | 0.5 | 16 | 0.047 | 1.5 |
| 30 | KNY_NGAMR33 | 60442 | 2016 | Nyanza | Male | 20-29 | <0.016 | <0.016 | 3 | 32 | 2 | 0.25 | 4 |
| 31 | KNY_NGAMR35 | 60525 | 2013 | Nairobi | Female | 20-29 | NT | NT | >256 | 48 | 32 | 0.125 | NT |
| 32 | KNY_NGAMR41 | 60526 | 2018 | Nyanza | Male | 40+ | <0.016 | <0.016 | 32 | 12 | 16 | NT | 4 |
| 33 | KNY_NGAMR50 | 60528 | 2018 | Nairobi | Male | 20-29 | <0.016 | <0.016 | 16 | 16 | 1.5 | 0.064 | 4 |
| 34 | KNY_NGAMR53 | 60530 | 2018 | Rift Valley | Male | 30-39 | <0.016 | <0.016 | 6 | 6 | 3 | 0.19 | 4 |
| 35 | KNY_NGAMR54 | 60531 | 2018 | Rift Valley | Male | 30-39 | <0.016 | <0.016 | 6 | 2 | 2 | 0.125 | 6 |
|  |  | NT-Not tested, CFX- cefixime, CRO- ceftriaxone, PEN- penicillin, TET-tetracycline, CIP-ciprofloxacin, AZM-azithromycin. | | | | | | | | | | | |
