# Supplementary material for: Genomic diversity of Neisseria gonorrhoeae Isolates in Kenya revealed by MLST, NG-MAST, and NG-STAR typing
Source: PLoS One. 2026 May 19;21(5):e0335831. doi: 10.1371/journal.pone.0335831 (PMC13186387; doi:10.1371/journal.pone.0335831)
Supplement: S2 Table — (DOCX) [file pone.0335831.s002.docx]

**S2 Table. EUCAST v8.0 (2018) Breakpoints for *N. gonorrhoeae***

| **Antibiotic** | **Susceptible (S)** | **Resistant (R)** |
| --- | --- | --- |
| **Ceftriaxone (CRO)** | ≤ 0.125 mg/L | > 0.125 mg/L |
| **Cefixime (CFX)** | ≤ 0.125 mg/L | > 0.125 mg/L |
| **Azithromycin (AZM)** | ≤ 0.25 mg/L | > 0.5 mg/L |
| **Ciprofloxacin (CIP)** | ≤ 0.03 mg/L | > 0.06 mg/L |
| **Tetracycline (TET)** | ≤ 0.5 mg/L | > 1 mg/L |
| **Penicillin (PEN)** | ≤ 0.06 mg/L | > 1 mg/L |
| **Spectinomycin (SPT)** | ≤ 64 mg/L | > 64 mg/L |
| **Gentamicin (GEN)** | *No clinical breakpoints* | *No clinical breakpoints* |
