# Supplementary material for: Genomic diversity of Neisseria gonorrhoeae Isolates in Kenya revealed by MLST, NG-MAST, and NG-STAR typing
Source: PLoS One. 2026 May 19;21(5):e0335831. doi: 10.1371/journal.pone.0335831 (PMC13186387; doi:10.1371/journal.pone.0335831)
Supplement: S3 Table — This table provides a cross-reference for isolates shown in Figure 2, allowing readers to easily match strain numbers to the PubMLST IDs and sequence type designations. Novel sequence types (STs) identified in this study are indicated in bold italics. (DOCX) [file pone.0335831.s003.docx]

S3 Table. Sequential numbering (1-35) of the study isolates with their corresponding PubMLST identifiers and assigned sequence types across the three typing schemes (MLST, NG-MAST, and NG-STAR). This table provides a cross-reference for isolates shown in Figure 2, allowing readers to easily match strain numbers to the PubMLST IDs and sequence type designations. Novel sequence types (STs) identified in this study are indicated in bold italics.

|  | **Isolate** | **PubMLST IDs** | **NG-MAST** | **NG STAR** | **MLST** |
| --- | --- | --- | --- | --- | --- |
| 1 | KNY_NGAMR1 | 60436 | ***19254*** | 2661 | ***13613*** |
| 2 | KNY_NGAMR2 | 60501 | ***19265*** | 2050 | 1928 |
| 3 | KNY_NGAMR3 | 60502 | ***19266*** | 1054 | 1893 |
| 4 | KNY_NGAMR4 | 60503 | ***19267*** | 2664 | 1588 |
| 5 | KNY_NGAMR5 | 60504 | ***19167*** | 2665 | 1599 |
| 6 | KNY_NGAMR6 | 60505 | ***19268*** | 1271 | 11367 |
| 7 | KNY_NGAMR7 | 60506 | ***18599*** | 1516 | 11366 |
| 8 | KNY_NGAMR8 | 60507 | ***19258*** | 1586 | 11365 |
| 9 | KNY_NGAMR9 | 59511 | ***19264*** | 2660 | ***13614*** |
| 10 | KNY_NGAMR10 | 60508 | 10134 | ***3186*** | 1932 |
| 11 | KNY_NGAMR11 | 60509 | ***19269*** | 1586 | 11365 |
| 12 | KNY_NGAMR13 | 60519 | 355 | 1586 | ***13782*** |
| 13 | KNY_NGAMR14 | 60511 | ***19270*** | 1255 | 11242 |
| 14 | KNY_NGAMR15 | 60516 | ***19087*** | 2170 | ***13782*** |
| 15 | KNY_NGAMR16 | 61330 | ***19263*** | 1890 | ***13766*** |
| 16 | KNY_NGAMR17 | 60513 | ***19271*** | 2667 | 1921 |
| 17 | KNY_NGAMR18 | 60514 | ***19168*** | 2668 | 8133 |
| 18 | KNY_NGAMR19 | 60515 | ***19169*** | 2669 | 1893 |
| 19 | KNY_NGAMR20 | 60512 | ***19255*** | 2666 | ***13780*** |
| 20 | KNY_NGAMR21 | 60517 | 11752 | 1586 | 11976 |
| 21 | KNY_NGAMR22 | 60518 | ***19168*** | 2668 | 8133 |
| 22 | KNY_NGAMR23 | 60510 | ***19255*** | 2666 | ***13780*** |
| 23 | KNY_NGAMR24 | 60520 | ***19168*** | 2668 | 8133 |
| 24 | KNY_NGAMR26 | 60439 | ***19261*** | 1890 | ***13779*** |
| 25 | KNY_NGAMR28 | 60521 | ***19260*** | ***3184*** | 1932 |
| 26 | KNY_NGAMR29 | 60522 | ***19262*** | ***3179*** | 11750 |
| 27 | KNY_NGAMR30 | 60441 | ***19259*** | 2170 | ***13763*** |
| 28 | KNY_NGAMR31 | 60523 | ***19272*** | ***3183*** | 1932 |
| 29 | KNY_NGAMR32 | 60524 | ***19170*** | 1603 | ***13782*** |
| 30 | KNY_NGAMR33 | 60442 | ***19166*** | 1890 | ***13764*** |
| 31 | KNY_NGAMR35 | 60525 | 10134 | 1271 | 1932 |
| 32 | KNY_NGAMR41 | 60526 | ***19168*** | 2668 | 8133 |
| 33 | KNY_NGAMR50 | 60528 | ***19256*** | ***3185*** | 1932 |
| 34 | KNY_NGAMR53 | 60530 | ***19262*** | ***3179*** | 11750 |
| 35 | KNY_NGAMR54 | 60531 | ***19257*** | ***3182*** | 8111 |
