# Supplementary material for: Genomic diversity of Neisseria gonorrhoeae Isolates in Kenya revealed by MLST, NG-MAST, and NG-STAR typing
Source: PLoS One. 2026 May 19;21(5):e0335831. doi: 10.1371/journal.pone.0335831 (PMC13186387; doi:10.1371/journal.pone.0335831)
Supplement: S4 Table — Novel sequence types (STs) identified in this study are indicated in bold italics. (DOCX) [file pone.0335831.s004.docx]

S4 Table. Identified MLST alleles and sequence types. *Novel sequence types (STs) identified in this study are indicated in bold italics*

| **Isolate** | **Region** | **Year of isolation** | ***abcZ*** | ***adk*** | ***aroE*** | ***fumC*** | ***gdh*** | ***pdhC*** | ***pgm*** | **MLST** |
| --- | --- | --- | --- | --- | --- | --- | --- | --- | --- | --- |
| KNY_NGAMR1 | Nairobi | 2015 | 59 | 39 | 170 | 237 | 148 | 153 | 65 | ***13613*** |
| KNY_NGAMR2 | Nairobi | 2015 | 126 | 39 | 67 | 111 | 149 | 153 | 65 | 1928 |
| KNY_NGAMR3 | Nairobi | 2015 | 59 | 39 | 67 | 157 | 147 | 153 | 133 | 1893 |
| KNY_NGAMR4 | Coast | 2016 | 59 | 39 | 67 | 158 | 148 | 71 | 65 | 1588 |
| KNY_NGAMR5 | Nyanza | 2016 | 59 | 39 | 67 | 157 | 148 | 153 | 65 | 1599 |
| KNY_NGAMR6 | Coast | 2017 | 59 | 39 | 67 | 111 | 189 | 153 | 65 | 11367 |
| KNY_NGAMR7 | Coast | 2014 | 59 | 39 | 67 | 111 | 147 | 71 | 65 | 11366 |
| KNY_NGAMR8 | Nyanza | 2013 | 59 | 112 | 67 | 158 | 148 | 71 | 65 | 11365 |
| KNY_NGAMR9 | Nyanza | 2016 | 59 | 112 | 67 | 111 | 189 | 153 | 65 | ***13614*** |
| KNY_NGAMR10 | Nyanza | 2016 | 59 | 39 | 67 | 78 | 189 | 153 | 65 | 1932 |
| KNY_NGAMR11 | Nyanza | 2014 | 59 | 112 | 67 | 158 | 148 | 71 | 65 | 11365 |
| KNY_NGAMR13 | Rift Valley | 2015 | 59 | 112 | 937 | 158 | 148 | 71 | 65 | ***13782*** |
| KNY_NGAMR14 | Rift Valley | 2014 | 59 | 39 | 170 | 78 | 189 | 153 | 65 | 11242 |
| KNY_NGAMR15 | Nyanza | 2015 | 59 | 112 | 937 | 158 | 148 | 71 | 65 | ***13782*** |
| KNY_NGAMR16 | Nyanza | 2015 | 109 | 39 | 67 | 157 | 150 | 153 | 133 | ***13766*** |
| KNY_NGAMR17 | Nyanza | 2015 | 59 | 39 | 67 | 111 | 148 | 71 | 65 | 1921 |
| KNY_NGAMR18 | Nyanza | 2015 | 59 | 39 | 170 | 158 | 148 | 71 | 65 | 8133 |
| KNY_NGAMR19 | Nyanza | 2015 | 59 | 39 | 67 | 157 | 147 | 153 | 133 | 1893 |
| KNY_NGAMR20 | Nyanza | 2016 | 59 | 112 | 67 | 158 | 147 | 71 | 65 | ***13780*** |
| KNY_NGAMR21 | Nyanza | 2016 | 59 | 112 | 67 | 158 | 148 | 153 | 65 | 11976 |
| KNY_NGAMR22 | Nyanza | 2017 | 59 | 39 | 170 | 158 | 148 | 71 | 65 | 8133 |
| KNY_NGAMR23 | Nyanza | 2014 | 59 | 112 | 67 | 158 | 147 | 71 | 65 | ***13780*** |
| KNY_NGAMR24 | Nyanza | 2016 | 59 | 39 | 170 | 158 | 148 | 71 | 65 | 8133 |
| KNY_NGAMR26 | Nyanza | 2016 | 109 | 39 | 67 | 983 | 150 | 153 | 65 | ***13779*** |
| KNY_NGAMR28 | Nyanza | 2017 | 59 | 39 | 67 | 78 | 189 | 153 | 65 | 1932 |
| KNY_NGAMR29 | Nyanza | 2017 | 109 | 39 | 67 | 157 | 150 | 153 | 65 | 11750 |
| KNY_NGAMR30 | Nyanza | 2017 | 59 | 112 | 67 | 157 | 148 | 71 | 65 | ***13763*** |
| KNY_NGAMR31 | Nyanza | 2017 | 59 | 39 | 67 | 78 | 189 | 153 | 65 | 1932 |
| KNY_NGAMR32 | Nyanza | 2017 | 59 | 112 | 937 | 158 | 148 | 71 | 65 | ***13782*** |
| KNY_NGAMR33 | Nyanza | 2016 | 109 | 39 | 67 | 157 | 150 | 901 | 133 | ***13764*** |
| KNY_NGAMR35 | Nairobi | 2013 | 59 | 39 | 67 | 78 | 189 | 153 | 65 | 1932 |
| KNY_NGAMR41 | Nyanza | 2018 | 59 | 39 | 170 | 158 | 148 | 71 | 65 | 8133 |
| KNY_NGAMR50 | Nairobi | 2018 | 59 | 39 | 67 | 78 | 189 | 153 | 65 | 1932 |
| KNY_NGAMR53 | Rift Valley | 2018 | 109 | 39 | 67 | 157 | 150 | 153 | 65 | 11750 |
| KNY_NGAMR54 | Rift Valley | 2018 | 126 | 39 | 170 | 158 | 148 | 71 | 65 | 8111 |
