# Supplementary material for: Genomic diversity of Neisseria gonorrhoeae Isolates in Kenya revealed by MLST, NG-MAST, and NG-STAR typing
Source: PLoS One. 2026 May 19;21(5):e0335831. doi: 10.1371/journal.pone.0335831 (PMC13186387; doi:10.1371/journal.pone.0335831)
Supplement: S5 Table — Novel sequence types (STs) identified in this study are indicated in bold italics. (DOCX) [file pone.0335831.s005.docx]

S5 Table. Identified NG-MAST alleles and sequence types. *Novel sequence types (STs) identified in this study are indicated in bold italics*

| **Isolate** | **Region** | **Year of isolation** | ***porB*** | ***tbpB*** | **NG-MAST** |
| --- | --- | --- | --- | --- | --- |
| KNY_NGAMR1 | Nairobi | 2015 | 90 | 2905 | ***19254*** |
| KNY_NGAMR2 | Nairobi | 2015 | 11156 | 362 | ***19265*** |
| KNY_NGAMR3 | Nairobi | 2015 | 11157 | 1807 | ***19266*** |
| KNY_NGAMR4 | Coast | 2016 | 11158 | 191 | ***19267*** |
| KNY_NGAMR5 | Nyanza | 2016 | 2824 | 1806 | ***19167*** |
| KNY_NGAMR6 | Coast | 2017 | 11159 | 9 | ***19268*** |
| KNY_NGAMR7 | Coast | 2014 | 121 | 726 | ***18599*** |
| KNY_NGAMR8 | Nyanza | 2013 | 970 | 2907 | ***19258*** |
| KNY_NGAMR9 | Nyanza | 2016 | 11155 | 9 | ***19264*** |
| KNY_NGAMR10 | Nyanza | 2016 | 2298 | 726 | 10134 |
| KNY_NGAMR11 | Nyanza | 2014 | 11160 | 2908 | ***19269*** |
| KNY_NGAMR13 | Rift Valley | 2015 | 90 | 133 | 355 |
| KNY_NGAMR14 | Rift Valley | 2014 | 11161 | 145 | ***19270*** |
| KNY_NGAMR15 | Nyanza | 2015 | 123 | 133 | ***19087*** |
| KNY_NGAMR16 | Nyanza | 2015 | 5286 | 2911 | ***19263*** |
| KNY_NGAMR17 | Nyanza | 2015 | 11162 | 5 | ***19271*** |
| KNY_NGAMR18 | Nyanza | 2015 | 5746 | 29 | ***19168*** |
| KNY_NGAMR19 | Nyanza | 2015 | 3440 | 612 | ***19169*** |
| KNY_NGAMR20 | Nyanza | 2016 | 90 | 2909 | ***19255*** |
| KNY_NGAMR21 | Nyanza | 2016 | 90 | 190 | 11752 |
| KNY_NGAMR22 | Nyanza | 2017 | 5746 | 29 | ***19168*** |
| KNY_NGAMR23 | Nyanza | 2014 | 90 | 2909 | ***19255*** |
| KNY_NGAMR24 | Nyanza | 2016 | 5746 | 29 | ***19168*** |
| KNY_NGAMR26 | Nyanza | 2016 | 5286 | 2906 | ***19261*** |
| KNY_NGAMR28 | Nyanza | 2017 | 4263 | 726 | ***19260*** |
| KNY_NGAMR29 | Nyanza | 2017 | 5286 | 2910 | ***19262*** |
| KNY_NGAMR30 | Nyanza | 2017 | 2855 | 133 | ***19259*** |
| KNY_NGAMR31 | Nyanza | 2017 | 11163 | 145 | ***19272*** |
| KNY_NGAMR32 | Nyanza | 2017 | 9388 | 191 | ***19170*** |
| KNY_NGAMR33 | Nyanza | 2016 | 5286 | 60 | ***19166*** |
| KNY_NGAMR35 | Nairobi | 2013 | 2298 | 726 | 10134 |
| KNY_NGAMR41 | Nyanza | 2018 | 5746 | 29 | ***19168*** |
| KNY_NGAMR50 | Nairobi | 2018 | 90 | 726 | ***19256*** |
| KNY_NGAMR53 | Rift Valley | 2018 | 5286 | 2910 | ***19262*** |
| KNY_NGAMR54 | Rift Valley | 2018 | 139 | 2051 | ***19257*** |
