# Supplementary material for: Genomic diversity of Neisseria gonorrhoeae Isolates in Kenya revealed by MLST, NG-MAST, and NG-STAR typing
Source: PLoS One. 2026 May 19;21(5):e0335831. doi: 10.1371/journal.pone.0335831 (PMC13186387; doi:10.1371/journal.pone.0335831)
Supplement: S6 Table — Novel sequence types (STs) identified in this study are indicated in bold italics. (DOCX) [file pone.0335831.s006.docx]

S6 Table. Identified NG-STAR alleles and sequence types. *Novel sequence types (STs) identified in this study are indicated in bold italics*

| **Isolate** | **Region** | ***mtrR*** | ***penA*** | **23S_rRNA** | ***gyrA*** | ***parC*** | ***ponA*** | ***porB*** | **NG STAR** |
| --- | --- | --- | --- | --- | --- | --- | --- | --- | --- |
| KNY_NGAMR1 | Nairobi | 57 | 294 | 100 | 1 | 2 | 100 | 13 | 2661 |
| KNY_NGAMR2 | Nairobi | 19 | 23 | 100 | 100 | 2 | 1 | 4 | 2050 |
| KNY_NGAMR3 | Nairobi | 10 | 285 | 100 | 1 | 49 | 1 | 100 | 1054 |
| KNY_NGAMR4 | Coast | 54 | 228 | 100 | 100 | 1 | 100 | 3 | 2664 |
| KNY_NGAMR5 | Nyanza | 229 | 285 | 100 | 1 | 22 | 1 | 19 | 2665 |
| KNY_NGAMR6 | Coast | 10 | 23 | 100 | 1 | 22 | 100 | 100 | 1271 |
| KNY_NGAMR7 | Coast | 98 | 20 | 100 | 1 | 49 | 1 | 3 | 1516 |
| KNY_NGAMR8 | Nyanza | 10 | 294 | 100 | 7 | 7 | 1 | 13 | 1586 |
| KNY_NGAMR9 | Nyanza | 10 | 294 | 100 | 7 | 22 | 100 | 14 | 2660 |
| KNY_NGAMR10 | Nyanza | 343 | 23 | 100 | 1 | 22 | 100 | 100 | ***3186*** |
| KNY_NGAMR11 | Nyanza | 10 | 294 | 100 | 7 | 7 | 1 | 13 | 1586 |
| KNY_NGAMR13 | Rift Valley | 10 | 294 | 100 | 7 | 7 | 1 | 13 | 1586 |
| KNY_NGAMR14 | Rift Valley | 10 | 23 | 100 | 1 | 22 | 100 | 3 | 1255 |
| KNY_NGAMR15 | Nyanza | 10 | 294 | 100 | 7 | 7 | 1 | 14 | 2170 |
| KNY_NGAMR16 | Nyanza | 10 | 294 | 100 | 1 | 22 | 100 | 14 | 1890 |
| KNY_NGAMR17 | Nyanza | 18 | 20 | 100 | 1 | 49 | 1 | 100 | 2667 |
| KNY_NGAMR18 | Nyanza | 54 | 228 | 100 | 1 | 49 | 100 | 100 | 2668 |
| KNY_NGAMR19 | Nyanza | 230 | 294 | 100 | 1 | 49 | 1 | 100 | 2669 |
| KNY_NGAMR20 | Nyanza | 10 | 285 | 100 | 7 | 49 | 1 | 13 | 2666 |
| KNY_NGAMR21 | Nyanza | 10 | 294 | 100 | 7 | 7 | 1 | 13 | 1586 |
| KNY_NGAMR22 | Nyanza | 54 | 228 | 100 | 1 | 49 | 100 | 100 | 2668 |
| KNY_NGAMR23 | Nyanza | 10 | 285 | 100 | 7 | 49 | 1 | 13 | 2666 |
| KNY_NGAMR24 | Nyanza | 54 | 228 | 100 | 1 | 49 | 100 | 100 | 2668 |
| KNY_NGAMR26 | Nyanza | 10 | 294 | 100 | 1 | 22 | 100 | 14 | 1890 |
| KNY_NGAMR28 | Nyanza | 10 | 23 | 100 | 7 | 22 | 100 | 14 | ***3184*** |
| KNY_NGAMR29 | Nyanza | 10 | 294 | 100 | 1 | 111 | 1 | 14 | ***3179*** |
| KNY_NGAMR30 | Nyanza | 10 | 294 | 100 | 7 | 7 | 1 | 14 | 2170 |
| KNY_NGAMR31 | Nyanza | 10 | 23 | 100 | 1 | 22 | 100 | 13 | ***3183*** |
| KNY_NGAMR32 | Nyanza | 10 | 294 | 100 | 7 | 22 | 1 | 13 | 1603 |
| KNY_NGAMR33 | Nyanza | 10 | 294 | 100 | 1 | 22 | 100 | 14 | 1890 |
| KNY_NGAMR35 | Nairobi | 10 | 23 | 100 | 1 | 22 | 100 | 100 | 1271 |
| KNY_NGAMR41 | Nyanza | 54 | 228 | 100 | 1 | 49 | 100 | 100 | 2668 |
| KNY_NGAMR50 | Nairobi | 10 | 23 | 100 | 7 | 22 | 100 | 13 | ***3185*** |
| KNY_NGAMR53 | Rift Valley | 10 | 294 | 100 | 1 | 111 | 1 | 14 | ***3179*** |
| KNY_NGAMR54 | Rift Valley | 10 | 20 | 100 | 1 | 22 | 100 | 100 | ***3182*** |
