# Supplementary material for: Genomic diversity of Neisseria gonorrhoeae Isolates in Kenya revealed by MLST, NG-MAST, and NG-STAR typing
Source: PLoS One. 2026 May 19;21(5):e0335831. doi: 10.1371/journal.pone.0335831 (PMC13186387; doi:10.1371/journal.pone.0335831)
Supplement: Attachment — Submitted filename: Reviewer-2_comments_Kivata-et-al_PONE-D-25-55705.docx [file pone.0335831.s007.docx]

Comments on Kivata et al_PONE-D-25-55705
**Genomic diversity on *Neisseria gonorrhoeae* isolates in Kenya revealed by MLST, NG-MAST and NG-STAR typing**.
November 2025

This manuscript describes the presence of different types of *Neisseria gonorrhoeae* (NG) strains in 4 regions in Kenya using genomic typing, after obtaining full genome sequences. It is interesting to see what has been circulating in this African country, since in the present databases mostly Western/ European types are described. In this respect it is interesting to see Figure 4 where the Kenyan sequences are in a tree with other global sequences of NG.
Although this reviewer recognizes that it is a formidable job to obtain whole genome sequences of NG strains, it is a severe limitation that only 35 strains were included. Kenya is an very large country with >50 million inhabitants, so this sample can hardly be representative for what is circulating. Another limitation is that only samples from 2013 to 2018 (5 years) were included which is at least 7 years ago from the present. These limitations should be clearly mentioned, also in the abstract.

Some other major and minor points must be taken in consideration to further improve this manuscript.

Major Comments:

- The Results are based on WGS data from 35 samples. In many lines the types are given in percentages. For example in line 187/188 is stated that in 2015 , 44.4% (CI….) of isolates displayed a novel MLST type. Please give **the number** of isolates instead of the percentage, since in 2015 this number was only 9 (line 223). Please adjust where applicable (also in lines 224 to 228 for example) to mention the absolute numbers of isolates and not (only) the percentages.
- Figure 1: please present absolute numbers for novel types and not percentages. Again, the numbers are very small and not representative for the years.
- The S1 Table is mentioned later in the text (line 157) then the S2 Table. Please reverse the S1 and S2 Table numbers so they will appear in the right order.
- The sentence on ciprofloxacin resistance (lines 189-190) does not fit in the middle of the paragraph on MLST. Please mention this for example in the NG-STAR paragraph which deals with antibiotic resistance gene polymorphisms.
- In S3 Table can be seen that most (23/35) samples were derived from the Nyanza region. So the finding that the two ST8133 types belonged to the Nyanza region is of no surprise. The other two each ‘region-based’ types ST-11365 and ST-13780 that were also from Nyanza is what can be expected by chance if so many strains were from that region. So the suggestion that there is clustering of MLSTs based on the region is not justified. The same comment goes for the NG-MAST types in lines 205-207. Please rephrase this, and avoid ‘region based’.
- S6 Table where relations are tested as ‘Group_vs_other’ is incomprehensible . I suggest to leave out this paragraph (lines 235 -243) and Table.
- In Figure 2 the Pub MLST IDs of the strains are difficult to read. You may consider to provide a supplementary table with numbers 1 to 35 in which per strain number each Pub MLST ID type number (MLST, NG-STAR- NG-Mast) is given. In that way all data on these strains will be more easily seen in the figure. Lines 268-270: I suggest that there actually is no Cluster 4; these strains are genetically too far apart.
- Line 285: The NG-STAR STs are mentioned in Figure 3. However, in this Figure the concatenated sequences of NG-STAR are shown. So the NG-STAR type itself is not needed and redundant information. Please adjust.
- The Discussion is very lengthy (5 pages) and contains both redundancies and textbook information. It should be reduced to 3 pages or less. For example: lines 374 – 376: this is a repetition of the results.; lines 429 (starting with ‘A small’ ) to 437 (up to ‘novelty’) is too general: text book stuff that can be deleted; lines 440-447: repetition of earlier text: delete; lines 453- 456 are redundant; Conclusions and recommendations: this can be shortened to two sentences.

Minor comments

- Line 40: please adjust to ‘suggests’.
- Line 77: please adjust to ‘porin B’ and ‘490 bp’.
- Line 88: please adjust to ‘strain evolution’.
- Line 114: can you provide a reference for the STI surveillance programme?
- Line 283: please adjust to ‘2 isolates’.
- Line 304 (in legend of Fig 3): please delete ‘formed’ before ‘comprised’.
- Line 330: please add ‘a’ before ‘variety’.
- Line 335: please replace ‘most’ by ‘highest’.
- Line 337: please adjust to ‘Western Australia’.
- Line 347: could the ‘stabilization’ of new types in 2018 possibly be due to the fact that sequences in PubMLST also originated around the year 2018?
- Line 370: please add ‘the’ before ‘present’.
- Line 373: please add ‘a’ before ‘heterogeneous’.
- Line 377: please adjust to ‘types’.
- Line 380: please add ‘a’ before ‘specific’.
- Line 382: please adjust to ‘azithromycin’ (without a capital A).
