# Supplementary material for: Genomic diversity of Neisseria gonorrhoeae Isolates in Kenya revealed by MLST, NG-MAST, and NG-STAR typing
Source: PLoS One. 2026 May 19;21(5):e0335831. doi: 10.1371/journal.pone.0335831 (PMC13186387; doi:10.1371/journal.pone.0335831)
Supplement: Attachment — Submitted filename: Response to Reviewers.docx [file pone.0335831.s009.docx]

**Date: 2^nd^ April, 2026**

**To:**	Sylvia Maria Bruisten, Ph.D,

Academic Editor

PLOS ONE

**From:**	Mary Wandia Kivata,

School of Pure And applied Sciences,

Department of Biological and Physical Sciences

Dear Editor,

Thank you for the opportunity to respond to the reviewers’ comments on our manuscript entitled “Genomic Diversity of *Neisseria gonorrhoeae* Isolates in Kenya Revealed by MLST, NG-MAST, and NG-STAR Typing”. We appreciate the reviewers’ efforts and have carefully considered their feedback. Below, we provide a detailed rebuttal to the points raised.

**Editor Comments:**

When submitting your revision, we need you to address these additional requirements.

1. Please ensure that your manuscript meets PLOS ONE's style requirements, including those for file naming.

***Response****: style requirements met*

2. Please provide additional details regarding participant consent. In the ethics statement in the Methods and online submission information, please ensure that you have specified (1) whether consent was informed and (2) what type you obtained (for instance, written or verbal, and if verbal, how it was documented and witnessed). If your study included minors, state whether you obtained consent from parents or guardians. If the need for consent was waived by the ethics committee, please include this information.

If you are reporting a retrospective study of medical records or archived samples, please ensure that you have discussed whether all data were fully anonymized before you accessed them and/or whether the IRB or ethics committee waived the requirement for informed consent. If patients provided informed written consent to have data from their medical records used in research, please include this information.

***Response:*** *Consent to participate was not applicable for this study because it was retrospective laboratory based, used archived samples, and there was no interaction with subjects. All data were fully anonymized before we accessed them and the WRAIR IRB qualified the research as not involving human subjects. (Page 8, lines 173-176)*

3. In the online submission form you indicate that your data is not available for proprietary reasons and have provided a contact point for accessing this data. Please note that your current contact point is a co-author on this manuscript. According to our Data Policy, the contact point must not be an author on the manuscript and must be an institutional contact, ideally not an individual. Please revise your data statement to a non-author institutional point of contact, such as a data access or ethics committee, and send this to us via return email. Please also include contact information for the third-party organization, and please include the full citation of where the data can be found.

**Response:** *Contact information has been changed to:*

KENYA MEDICAL RESEARCH INSTITUTE (KEMRI)

SCIENTIFIC AND ETHICS REVIEW UNIT(SERU)

P.O. BOX 54840 00200 OFF MBAGATHI ROAD,

NAIROBI, KENYA

HOUSE NUMBER 8, KEMRI HEADQUARTERS.

Office Mobile: 0717 719 477

Extension Number: 3333 or 3332

Email: seru@kemri.go.ke and kemriseru18@gmail.com

Data on the online availability of the generated sequence has been shown in page 6 line 117-118

4. We notice that your supplementary tables are included in the manuscript file. Please remove them and upload them with the file type 'Supporting Information'. Please ensure that each Supporting Information file has a legend listed in the manuscript after the references list.

***Response:*** *The supporting files information have been removed from the manuscript file and uploaded separately as recommended (page 23)*

5. If the reviewer comments include a recommendation to cite specific previously published works, please review and evaluate these publications to determine whether they are relevant and should be cited. There is no requirement to cite these works unless the editor has indicated otherwise.

***Response:*** *Noted*

**Reviewers' comments:**

**Reviewer #1:**

A summary of the genetic variability of *Neisseria gonorrhoeae* in an under-researched region. A limited sample size, but a thorough analysis.

**Major Comments:**

1. Results: I would like to see a summary of the sample demographics in your results. I know you listed all of the data in your supplemental files, but it is nice to see it summarized as well. Counts of how many isolates come from each region (which is relevant later on when you discuss regional differences) and how many per year, as well as age and gender distributions.

***Response:*** *A paragraph describing the sample demographics has been included in the results section (Page 9, lines 179 -189)*

2. Lines 198-200: The wording here is somewhat unclear and the information in this section could be written more concisely. An example of how this may be reworded is: Thirty-one (88.6%) isolates belonged to 26 novel NG-MAST STs, with 2 isolates each of STs and one isolate each of STs. Four isolates belonged to 3 known STs: ST-355 (n=?), ST-10134 (n=?), and ST-11752 (n=?). The most common ST was ST-19168, found in four isolates (11.4%) from Nyanza, all of which were also MLST ST-8133.

***Response:*** *The section has been rephrased to bring clarity (Page 10, lines 206-212)*

3. Lines 343-358: Could the underrepresentation of Kenyan sequences in the various MLST databases be contributing to the large number of novel STs? Particularly with regard to the stabilization trend amongst the 2018 isolates. This does not appear to me to be a notable trend as the n value is very low and there are too many confounding factors. Furthermore, the presence of novel NG-STAR STs in 2017-2018 is likely to be an artefact of limited representation of African strains in this database. Inference of lineage stabilization and genomic diversity requires comprehensive databases with sufficient regional representation as well as a large sample size. Rather than focusing on temporal changes in diversity here, it may be more valuable to highlight the importance of including regionally diverse strains in international databases to effectively capture the true diversity of *Neisseria gonorrhoeae* as well as allow for the effective study of genomic diversity changes worldwide.

***Response:*** *Thank you for this insightful observation. We have revised the discussion to emphasize the importance of expanding regional representation in global databases (Page 16, lines 361-372)*

**Minor Comments:**

1. Line 55: There is an unnecessary comma here

***Response:*** *Comma deleted (Page 3, line 52)*

2. Line 129: Version 1.8 is no longer available and the website is now hosted at https://cge.food.dtu.dk/services/MLST/. Why did you use this program rather than pubMLST to determine the ST types? Lines 131-135: www.ng-mast.net is no longer functional. The NG-MAST database is now being maintained by pubMLST. Lines 136-138: While pubMLST does host a version of NG-STAR, the main host for the database is <https://ngstar.canada.ca/>

***Response for the three comments:*** *Thank you for these helpful clarifications. At the time of our analyses, MLST version 1.8 hosted at the Centre for Genomic Epidemiology was widely used and provided a streamlined workflow for batch processing of assembled genomes. Similarly, NG-MAST typing was conducted using the www.ng-mast.net database, which at the time was the standard resource for allele assignment and sequence type determination. While NG-STAR typing was performed using the scheme implemented in PubMLST, we recognize that the primary host for the NG-STAR database is now https://ngstar.canada.ca/.*

*Clarifications have been added in the Methods section to ensure readers are aware of the current database location and to guide future analyses toward the most up-to-date resources. (Page 6&7, lines 120-139)*

3. Lines 188-189: This sentence doesn’t seem to belong here, as it’s unrelated to MLST typing

***Response:*** *Thank you for the observation. The sentence has been deleted (Page 9, lines 198 - 199)*

4. Lines 193-194: There are many more isolates from the Nyanza region, so it is unsurprising for some STs to be found there and nowhere else - please be sure to include the uneven distribution of isolates by region in your discussion.

***Response:*** *We agree that the with the observation. This has been clarified in the discussion section (Page 18, lines 438-442)*

5. Lines 198-200: The wording here is somewhat unclear. An example of how this may be reworded is: Thirty-one (88.6%) isolates belonged to 26 novel NG-MAST STs, with 2 isolates each of STs and one isolate each of STs. Four isolates belonged to 3 known STs: ST-355 (n=?), ST-10134 (n=?), and ST-11752 (n=?). The most common ST was ST-19168, found in four isolates (11.4%) from Nyanza, all of which were also MLST ST-8133.

***Response:*** *This comment has been addressed under the major comments. Please see response to the second major comment above.*

6. Lines 217-218: This sentence is unclear. Do you mean, “Two isolates, KNY_NGAMR20 and KNY_NGAMR23, from Nyanza shared the same NG-STAR, NG-MAST and MLST types”?

***Response:*** *The sentence has been rephrased (Page 11, lines 225-226)*

7. Lines 222-223: Please add a denominator to your n values for each year. It is difficult to understand the context of the number of novel STs without knowing the number of samples in total per year.

***Response:*** *The denominators have been added as recommended and the whole section revised. (Page 11, line 231)*

8. Line 330: Please change to “…identified a variety of…”

***Response:*** *Introduction paragraph of the discussion has been rephrased (Page 15, lines 351-357)*

9. Line 337: Please change to “…Western Australia”

***Response:*** *Change effected (Page 15, line 357)*

10. Lines 339-340: Previous studies have demonstrated that NG-MAST has a higher discriminatory power compared with MLST. Eg: <https://pmc.ncbi.nlm.nih.gov/articles/PMC3811299/>

***Response:*** *We agree with the reviewer’s observation and have revised the manuscript to acknowledge prior evidence supporting NG‑MAST’s superior discriminatory power (Page15, line 355-357)*

11. Line 373: Please change to “…belong to a heterogenous population”

***Response:*** *Change effected (Page 18, line 435)*

12. Lines 374-376: This sentence is unclear

***Response:*** *This section has been rewritten to enhance clarity (Page 18, line 430 - 433)*

13. Line 466: AMR should be defined at the beginning of the article, not at the end

***Response:*** *The section has been rewritten and abbreviation deleted (Page 20, line 469)*

**Reviewer #2:**

This manuscript describes the presence of different types of *Neisseria gonorrhoeae* (NG) strains in 4 regions in Kenya using genomic typing, after obtaining full genome sequences. It is interesting to see what has been circulating in this African country, since in the present databases mostly Western/ European types are described. In this respect it is interesting to see Figure 4 where the Kenyan sequences are in a tree with other global sequences of NG.

Although this reviewer recognizes that it is a formidable job to obtain whole genome sequences of NG strains, it is a severe limitation that only 35 strains were included. Kenya is a very large country with >50 million inhabitants, so this sample can hardly be representative for what is circulating. Another limitation is that only samples from 2013 to 2018 (5 years) were included which is at least 7 years ago from the present. These limitations should be clearly mentioned, also in the abstract.

***Response:*** *We acknowledge the reviewer’s concern regarding the limited number of isolates and the timeframe of sampling. Indeed, only 35 strains were included, collected between 2013 and 2018, which is a small dataset relative to Kenya’s population of over 50 million. This sample cannot be considered fully representative of the circulating gonococcal population nationwide. Furthermore, the isolates were obtained at least seven years prior to the present, and therefore may not reflect current strain diversity or resistance trends. These limitations have been clearly stated in the discussion section of the revised manuscript, to ensure transparency about the scope and generalizability of our findings (Page 19, line 438 - 442)*

Some other major and minor points must be taken in consideration to further improve this manuscript.

**Major Comments:**

1. The Results are based on WGS data from 35 samples. In many lines the types are given in percentages. For example, in line 187/188 is stated that in 2015, 44.4% (CI….) of isolates displayed a novel MLST type. Please give the number of isolates instead of the percentage, since in 2015 this number was only 9 (line 223). Please adjust where applicable (also in lines 224 to 228 for example) to mention the absolute numbers of isolates and not (only) the percentages.

***Response:*** *The results section on sequence typing (Line 190) and temporal distribution of novel sequence types (line 228) have been rewritten to capture the recommended changes.*

2. Figure 1: please present absolute numbers for novel types and not percentages. Again, the numbers are very small and not representative for the years.

***Response:*** *Change effected inside the graph as suggested (Fig 1)*

3. The S1 Table is mentioned later in the text (line 157) then the S2 Table. Please reverse the S1 and S2 Table numbers so they will appear in the right order.

***Response:*** *S1 table is mentioned first in page 3, line 100. Then S2 in page 5, line 103*

4. The sentence on ciprofloxacin resistance (lines 189-190) does not fit in the middle of the paragraph on MLST. Please mention this for example in the NG-STAR paragraph which deals with antibiotic resistance gene polymorphisms.

***Response:*** *Thank you for the observation. The sentence has been deleted (Page 9, lines 198 - 199)*

5. In S3 Table can be seen that most (23/35) samples were derived from the Nyanza region. So, the finding that the two ST8133 types belonged to the Nyanza region is of no surprise. The other two each ‘region-based’ types ST-11365 and ST-13780 that were also from Nyanza is what can be expected by chance if so, many strains were from that region. So, the suggestion that there is clustering of MLSTs based on the region is not justified. The same comment goes for the NG-MAST types in lines 205-207. Please rephrase this, and avoid ‘region based’.

***Response:*** *We agree that the with the observation. This has been clarified in the discussion section (Page 18, lines 438-442)*

6. S6 Table where relations are tested as ‘Group_vs_other’ is incomprehensible. I suggest to leave out this paragraph (lines 235 -243) and Table.

***Response:*** *The section on “association between typing schemes and antimicrobial resistance” and S6 Table have been deleted. A sentence explaining the relationship between typing schemes and AMR has been added to the NGSTAR phylogeny section (Page 14, lines 305-306)*

7. In Figure 2 the Pub MLST IDs of the strains are difficult to read. You may consider to provide a supplementary table with numbers 1 to 35 in which per strain number each Pub MLST ID type number (MLST, NG-STAR- NG-Mast) is given. In that way all data on these strains will be more easily seen in the figure. Lines 268-270: I suggest that there actually is no Cluster 4; these strains are genetically too far apart.

**Response:** *To improve clarity, we have provided a supplementary table (S6 Table) listing all 35 isolates, numbered sequentially, with their corresponding PubMLST IDs and assigned sequence types (MLST, NG-MAST, and NG-STAR). The sentence describing cluster 4 has been rephrased based on the observation made by the reviewer (Page 12 & 13, lines 274-276)*

8. Line 285: The NG-STAR STs are mentioned in Figure 3. However, in this Figure the concatenated sequences of NG-STAR are shown. So, the NG-STAR type itself is not needed and redundant information. Please adjust.

***Response:*** *Thank you for this observation. We appreciate the concern regarding potential redundancy in Figure 3. While the phylogeny is indeed inferred from concatenated NG-STAR loci, we intentionally included NG-STAR ST labels to illustrate how specific STs cluster in relation to antimicrobial AMR profiles. This dual presentation allows readers to interpret both the evolutionary relationships based on concatenated loci and the epidemiological relevance of ST clustering, particularly in relation to resistance determinants. We believe retaining the NG-STAR STs in the figure provides added value by linking phylogenetic structure to practical surveillance markers.*

9. The Discussion is very lengthy (5 pages) and contains both redundancies and textbook information. It should be reduced to 3 pages or less. For example: lines 374 - 376: this is a repetition of the results.; lines 429 (starting with ‘A small’) to 437 (up to ‘novelty’) is too general: text book stuff that can be deleted; lines 440-447: repetition of earlier text: delete; lines 453- 456 are redundant; Conclusions and recommendations: this can be shortened to two sentences.

***Response:*** *The discussion and conclusion sections have been rewritten based on suggestions from the other reviewers and redundancy reduced as recommended.*

**Minor comments**

1. Line 40: please adjust to ‘suggests’.

***Response:*** *Change effected. Line 37*

2. Line 77: please adjust to ‘porin B’ and ‘490 bp’.

***Response:*** *Change effected. Line 70-71*

3. Line 88: please adjust to ‘strain evolution’.

***Response:*** *Change effected. Line 81*

4. Line 114: can you provide a reference for the STI surveillance programme?

***Response:*** *Change effected. Line 98*

5. Line 283: please adjust to ‘2 isolates.

***Response:*** *Change effected. Line 291*

6. Line 304 (in legend of Fig 3): please delete ‘formed’ before ‘comprised’.

***Response:*** *Change effected. Line 315*

7. Line 330: please add ‘a’ before ‘variety’.

***Response:*** *Introduction paragraph of the discussion has been rephrased (Page 15, lines 351-357)*

8. Line 335: please replace ‘most’ by ‘highest’.

***Response:*** *Introduction paragraph of the discussion has been rephrased (Page 15, lines 351-357)*

9. Line 337: please adjust to ‘Western Australia’.

***Response:*** *Change effected (Page 15, line 357)*

10. Line 347: could the ‘stabilization’ of new types in 2018 possibly be due to the fact that sequences in PubMLST also originated around the year 2018?

***Response:*** *Thank you for this insightful observation. We have revised the discussion to emphasize the importance of expanding regional representation in global databases (Page 16, lines 361-372)*

11. Line 370: please add ‘the’ before ‘present’.

***Response:*** *The paragraph has been rewritten (Page 18, lines 443)*

12. Line 373: please add ‘a’ before ‘heterogeneous’.

***Response:*** *Change effected (Page 18, line 435)*

13. Line 377: please adjust to ‘types’.

***Response:*** *The paragraph has been rewritten Change effected (Page 16, line 373-382)*

14. Line 380: please add ‘a’ before ‘specific’.

***Response:*** *The paragraph has been rewritten (Page 16, line 477)*

15. Line 382: please adjust to ‘azithromycin’ (without a capital A).

***Response:*** *Change effected through out the document.*

**Reviewer #3:**

1. The authors characterize 35 *Neisseria gonorrhoeae* strains from four different geographical locations in Kenya, dating from 2013-18. The methods used for characterization of these strains are those commonly used. The low number of strains, coupled with the fact that strains came from four different locations and were obtained over five years, is almost a guarantee for high diversity between strains.

The conclusion of the authors, “The lack of regional clustering and varied AMR profiles suggest widespread transmission of heterogeneous gonococcal populations”, is only partially supported by the findings, since the number of strains sequenced was very low.

A very recent publication from Mehta et al. (Genomic trends and emerging antimicrobial resistance in *Neisseria gonorrhoeae* over two decades in Kenya, Microbiol Spectr. 2025 Oct 7;13(11): e01586-25), accepted September 1st, 2025, describes a collection of 218 strains from Kenya, partly isolated between 2002-09 and partly between 2020-22. Although I am not completely sure of the date of submission of the manuscript from Kivata et al. and the publication date of the paper mentioned above, I think data from Kivata et al. should at least be compared to those of Mehta et al. as an addition to the manuscript.

***Response:*** *We thank the reviewer for bringing this very recent and relevant publication by Mehta et al. (2025) to our attention. We agree that a comparison with their findings significantly strengthens our manuscript by providing a broader temporal and geographical context for gonococcal genomic surveillance in Kenya. We have now incorporated a comparison with Mehta et al. (2025) in the Discussion section of our revised manuscript (Page 16 & 17 lines 383-399)*

*Specifically, we have noted that while our study focused on isolates from 2013–2018 across four regions (Nairobi, Coastal Kenya, Nyanza, and Rift Valley), Mehta et al. examined trends in Western Kenya (Kisumu) over two decades (2002–2009 and 2020–2022). Both studies highlight the high genetic diversity and the prevalence of legacy resistance (ciprofloxacin, penicillin, and tetracycline) in Kenyan gonococcal populations. Interestingly, both studies identified similar predominant lineages (e.g., MLST ST-8133 and ST-1932) and noted the emergence of novel sequence types. We have also contrasted our findings regarding azithromycin and cephalosporin susceptibility, noting that while Mehta et al. reported the emergence of azithromycin resistance and cephalosporin alert values in their 2020–2022 cohort, our 2013–2018 isolates remained largely susceptible, with only low-level azithromycin resistance observed.*

Other comments:

1. Figure 1 does not provide any relevant information, since the number of strains collected in some years was extremely low (2013 (n=2), 2014 (n=4), 2018 (n=4)). Peaks in this figure could easily be explained by one or two strains that are cause of differences. No statistics were done, but if these had been done, I would expect that a sentence like “NG-STAR displayed fewer novel profiles, appearing sporadically but rising substantially in 2017 (57.1%) and 2018 (75%)” might not be supported by such an analysis.

***Response:*** *We acknowledge the reviewer's concern regarding the small sample sizes in certain years (2013, 2014, and 2018) and the potential for these small numbers to skew the interpretation of temporal trends. We agree that the percentage-based peaks in these years are sensitive to individual isolates and may not represent statistically significant shifts in the population. In response to this feedback, we have made the following revisions:*

1. *Revised Figure 1: We have updated Figure 1 to include the absolute number of isolates (n) for each year on the x-axis to provide immediate context for the percentages shown.*
2. *Softened the Language: We have revised the text in the Results and Discussion sections to avoid overstating these trends. Specifically, we have replaced "rising substantially" with more cautious language and explicitly noted the limitations imposed by the small sample sizes in those years (page 11, lines, 229-241 and 362-370).*
3. *Added a Disclaimer: We have added a statement in the Results section (Page 11, lines 231-232) acknowledging that the high proportions of novelty in years with low sample sizes (e.g., 2018) should be interpreted as exploratory rather than definitive evidence of a population-wide shift.*

*We believe these changes address the reviewer's concern by providing a more transparent and conservative interpretation of the temporal data.*

2. It is unclear to me what was the base for selection of the strains included in fig.4 outside the strains cultured in the present study. Have they been isolated in the same years? I would rather suggest to include more strains from other African countries for comparison – since they seem more related to the strains from the present study – and a randomized small sample of strains from non-African countries.

***Response:*** *We appreciate the reviewer's constructive feedback regarding the selection of global reference strains in Figure 4. The initial selection of the 39 additional strains from PubMLST was intended to include globally recognized high-risk resistant lineages (e.g., MLST ST-1901, NG-MAST ST-1407) to demonstrate that the Kenyan isolates represent distinct, locally evolving lineages rather than recent introductions of pandemic clones. These reference strains were selected based on their clinical significance and availability in the PubMLST database, covering a broad temporal range to ensure a robust comparison. However, we agree with the reviewer that a more focused comparison with African strains would provide more relevant context, given the closer genetic relationship observed. In response to this suggestion, we have:*

1. *Revised the Reference Dataset: We have updated the global context analysis by including a larger proportion of N. gonorrhoeae sequences from other African countries available in PubMLST, particularly those isolated during a similar timeframe (2010–2020).*
2. *Refined the Non-African Sample: We have replaced the previous broad selection of non-African strains with a smaller, randomized sample of representative global lineages to maintain the global context without overshadowing the regional comparison.*
3. *Updated Figure 4: Figure 4 has been regenerated to reflect this more balanced dataset, providing a clearer picture of how Kenyan isolates relate to both regional and global gonococcal populations (Page 15, Lines 338- 348).*
4. *Clarified the Methodology: We have updated the Phylogeny analysis section (Page 7, Lines 148-155) to explicitly state the criteria for selecting these reference strains, including their geographic origin and isolation years.*

*We believe these revisions significantly improve the relevance of the phylogenetic comparison and address the reviewer's concerns.*

Minor comments:

1. p.4, l.67-68: I should not designate a typing method from 2017 “recently described”

***Response:*** *The words recently described have been deleted (Page3, line 62)*

2. The regions mentioned in figure 3 have different names from those in Materials and Methods and in table S1. Please use the same designation for regions throughout the manuscript.

***Response:*** *We thank the reviewer for this observation. We have clarified the regional designations in the revised manuscript to ensure consistency. Specifically, we have added a statement in the "Materials and Methods" section to explain that Kisumu and Kombewa are specific study sites located within the larger Nyanza region (Page 5, line 92 - 95)*

3. Figure 4 is difficult to read due to the use of colors. Strains from the present study are shown in green, but also some other strains are shown in a slightly different type of green. For optimal use of the different colors, I would rather suggest to give clearly distinct colors to strains from Kenya and from other African countries and to use only one color for strains from non-African countries – those are clearly distinct from almost all strains from Africa.

***Response:*** *Figure 4 has been regenerated to reflect more balanced dataset, providing a clearer picture of how Kenyan isolates relate to both regional and global gonococcal populations (Page 15, Lines 338- 348). Strains from Kenya and from other African countries have been assigned distinct colors and those from non-African countries are shown in black*

We sincerely thank the reviewers for their insightful and constructive feedback, which has greatly enhanced the quality and clarity of our manuscript. Their detailed comments on temporal trends, regional classifications, and phylogenetic comparisons were invaluable in refining our analysis. In response, we have incorporated more comprehensive regional comparisons and clarified our methodologies, thereby strengthening the robustness and relevance of our contribution to gonococcal genomic surveillance in Africa. We trust that our revisions and clarifications adequately address the concerns raised, and we respectfully request reconsideration of our manuscript for publication in *PLOS ONE*.

Yours faithfully,

Mary Wandia Kivata, PhD (Corresponding Author)

Department of Biological and Physical Sciences,

Karatina University, Karatina, Kenya

Email: [marywandia086@gmail.com/mkivata@karu.ac.ke](mailto:marywandia086@gmail.com/mkivata@karu.ac.ke)

**Co-authors**

Fredrick Lunyagi Eyase, Wallace Dimbuson Bulimo, Valerie Oundo, Esther Waguche, Wilton Mwema Mbinda and Margaret Mbuchi
